# Efficient visible light-induced degradation of rhodamine B by W(N_x_S_1−x_)_2_ nanoflowers

**DOI:** 10.1038/srep40784

**Published:** 2017-01-20

**Authors:** Peitao Liu, Jingyan Zhang, Daqiang Gao, Weichun Ye

**Affiliations:** 1 Key laboratory for magnetism and Magnetic Materials of MOE, Lanzhou University, Lanzhou 730000, P. R. China; 2 Department of Chemistry, Lanzhou University, Lanzhou 730000, P. R. China

## Abstract

Here, W(N_x_S_1−x_)_2_ nanoflowers were fabricated by simple sintering process. Photocatalytic activity results indicated our fabricated N-doped WS_2_ nanoflowers shown outstanding photoactivity of degradating of rhodamine B with visible light. Which is attributed to the high separation efficiency of photoinduced electron–hole pairs, the broadening of the valence band (VB), and the narrowing of energy band gap. Meanwhile, our work provided a novel method to induce surface sulfur vacancies in crystals by introduing impurities atoms for enhancing their photodegradation.

In the past decades, there has been a great interest in developing semiconductor-based photocatalysts due to its high catalytic efficiency and good stability for water splitting and removal of hazardous organic compounds in industrial wastewater using solar energy^1^,^2^,^3^,^4^,^5^,^6^. TiO_2_, a typical traditional photocatalyst, has many merits, including its low cost, high efficiency and excellent stability^7^. However, it can’t absorb visible light and suffers from fast recombination rate of the photogenerated charge carriers^8^. In order to overcome these drawbacks, numerous investigations have been devoted to give new types of photocatalysts, where two-dimensional (2D) nanomaterials with exotic electronic properties and high specific surface areas are considered to be the good candidates^9^,^10^, as well as, they have attracted tremendous attention in heterogeneous catalysis^11^,^12^,^13^, sensors^14^, energy storage^15^,^16^ and electronics^17^,^18^,^19^.

Recently, transition metal sulfide has attracted intensive attention for their graphene-like structure. Tungsten disulfide (WS_2_), belonging to layered transition-metal dichalcogenides family, exhibits extraordinary electrical^19^ and photonic properties^20^,^21^. WS_2_ possesses hexagonal crystal structure with space group P63/mmc and each WS_2_ monolayer contains an individual layer of W atoms with 6-fold coordination symmetry, which are then hexagonally packed between two trigonal atomic layers of S atoms^22^. Generally, bulk WS_2_ has an indirect band gap of 1.35 eV, and when it is thinned to a single layer it becomes direct band gap semiconductor with a gap of 2.05 eV^23^,^24^. Hence, fewer layers WS_2_ nanosheets are the promising candidates for photocatalyst because of the number of active sites increases with the specific surface area at the nanoscale and the sites promote interfacial charge transfer for photo-induced electron-hole pairs^25^,^26^.

Nitrogen (N) doping is widely used in traditional semiconductor industry for effectively controlling their electronic properties. Recently, results indicated that the N doped graphene had the improved photocatalytic performance of photocatalysts than the bare graphene. Sacco *et al*. found that the N-doped TiO_2_ showed a higher photocatalytic activity for photodegradation of phenol under visible light irradiation than the TiO_2_ and titanium dioxide (P25)^8^. Meng *et al*. also reported that the photocatalytic the MO (methyl orange) evolution of N-La_2_Ti_2_O_7_ could be effectively improved by N doping^27^. In addition, many other researchers also demonstrated that various photocatalysts such as N-ZrO_2_^28^, N-(BiO)_2_CO_3_^29^, N-BiVO_4_^30^ and N-ZnO^31^ showed a higher photocatalytic performance compared to their pure phase.

In this paper, we reported a different approach for the synthesis of WS_2_ nanoflowers with in-suit nitrogen-doping by a simple sintering process. Results indicated that the fabricated N-doped WS_2_ nanoflowers showed a BET area as high as 58.87 m^2^/g, which was 19.3 times than that of bare WS_2_ nanosheets (BET area 3.05 m^2^/g)^32^. In addition, we reported the excellent visible light-induced degradation of rhodamine B by N-doped WS_2_ nanoflowers. Results indicated that 20 mg of our photocatalysis could completely degrade 50 ml of 20 mg L^−1^ RhB in 70 minutes with excellent recycling and structural stability.

## Experiment

All of the starting reagents used in this research are of analytical purity and used without further purification.

**Figure 1:**
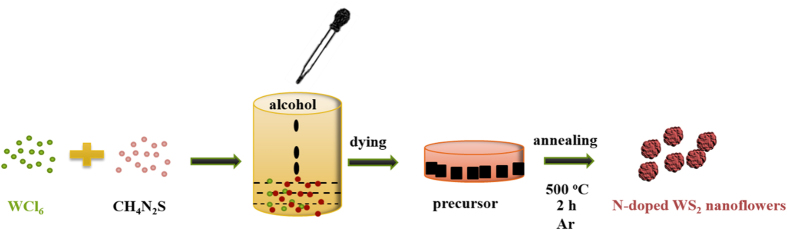
Schematic representation of the fabrication of W(N_x_S_1−x_)_2_ nanoflowers.

W(N_x_S_1−x_)_2_ nanoflowers were synthesized by an operability sintering method (as shown in Fig. 1). 0.5 g tungsten hexachloride (WCl_6_) was mixed with different amount of thiourea (CH_4_N_2_S) (0.5 g, 1 g, 2 g) by dropwise addition of alcohol. Then the dark-grey precursor powders were formed after drying and transferred into a quartz boat and heated in a tube furnace for 2 h under 0.1 L min^−1^ argon flow at 550 °C.

In order to further compare, bulk N-dope WS_2_ was prepared with the 0.5 g tungsten WCl_6_ mixed with 2 g CH_4_N_2_S by keeping above experiment condition in a tube furnace for 2 h under 0.1 L min^−1^ argon flow at 850 °C.

α-Fe_2_O_3_@ N-doped MoS_2_ heterostructures were synthesized by the hydrothermal method, where 90 mg N-doped MoS_2_ were dissolved into 32 ml deionized water. Then 0.202 g Fe(NO_3_)_3_·9H_2_O and 0.3 g CO(NH_2_)_2_ were dissolved into above solution under magnetic stirring. After that, 0.006 g sodium dodecyl benzenesulphonate (SDBS) were added into the above solution and continuous stirred in a water bath of 60 °C for 30 min. Finally, the solution was transferred to a 40 ml reactor and maintained at 90 °C for 12 h before being cooled down in air.

The crystal structure of the samples were measured by X-ray diffractometry (XRD) in a Philips/X’ Pert PRO diffractometer with Cu *Ka* radiation. Scanning electron microscope (SEM, Hitachi S-4800) and high resolution transmission electron microscope (HRTEM, TecnaiTM G2 F30, FEI, USA) were used to observe the morphology and structure of the products. In addition, X-ray photoelectron spectroscopy (XPS, VG Scientific ESCALAB-210) was employed to study the chemical nature of N, W and S with Al *Ka* X-ray, where the N concentration for the obtained three samples were measured to be 0.3 at.%, 0.6 at.% and 1.2 at.%. For convenience, the three samples were named as S 0.3, S 0.6, S 1.2. The Brunauer-Emmett-Teller (BET) surface area and pore width were measured by using a Micrometrics ASAP 2020 V403 measurement. Meanwhile, Raman spectra were measured in a room temperature using a Jobin-Yvon HR 800 spectrometer.

The photocatalytic activity of the samples were measured by degradation of RhB with a 175 W halogen lamp. 50 ml RhB (20 mg l^−1^) were placed in a glass, meanwhile, 20 mg photocatalysts were added under constantly stirring. Photocalytic activity of the sample was evaluated under visible light irradiation. At certain time intervals, 4 ml solution was taken out and using a centrifugal machine to remove photocatalytic. Then the filtrates were analyzed by recording variations of the absorption band maximum (553 nm) in the UV-vis spectra of RhB by using a UV-vis spectrophotometer. In addition, the recyclability of the sample was also investigated.

## Results and Discussion

### Characterization

**Figure 2:**
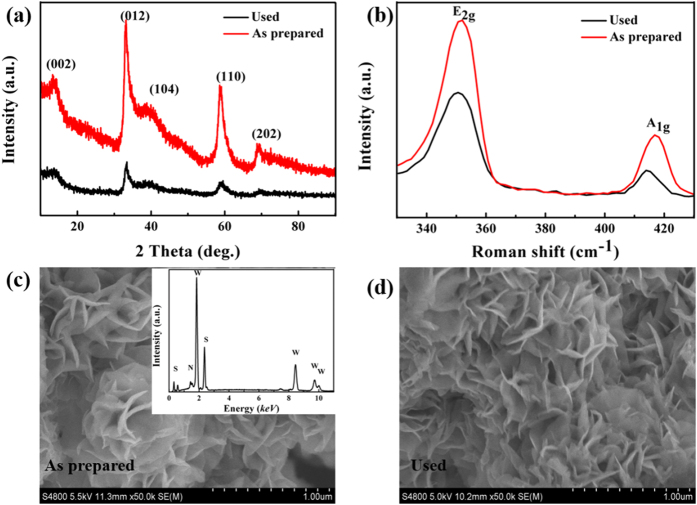
(**a**) XRD patterns, (**b**) Raman spectra, and (**c**,**d**) SEM images of S1.2 and the Used.

The obtained product of S1.2 and the **Used sample** (N-doped WS_2_ nanoflowers were used by the photocatalytic activity testing) were first measured by XRD and the results are illustrated in Fig. 1a. As can be seen that the five distinct peaks correspond to (002), (012), (104), (110), and (202) diffraction peacks of hexagonal WS_2_ (JCPDF 84–1399). For the **Used sample**, all the diffraction peaks exist and no other new phase appear, indicating that our sample has a stable structure in the photocatalytic process, which is further proved by Raman spectrum (Fig. 2b). The Raman spectrum shows typical features of layered WS_2_ where the 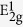 and A_1g_ modes are, located around 350 and 417 cm^−1 ^33,34. For the **Used sample**, the two distinct peaks were similar to the primitive product, providing more stable evidence for the property. To investigate the morphology of samples, the SEM measurement was considered and the result for sample S1.2 are presented in Fig. 2c and d. It can be seen from Fig. 2c and d that the sample show the flower-like structure and each of the component shows nanosheet feature. It can be seen that the morphology of our sample didn’t change obviously after photocatalytic, which also reveal the obtained product has a stable structure. Besides, energy-dispersive X-ray spectroscopy (EDS) analysis was carried out to verify the element-composition of the sample. As shown in the inset of Fig. 2c, EDS result clearly shows the presence of elements W, S and N in our fabricated sample.

**Figure 3:**
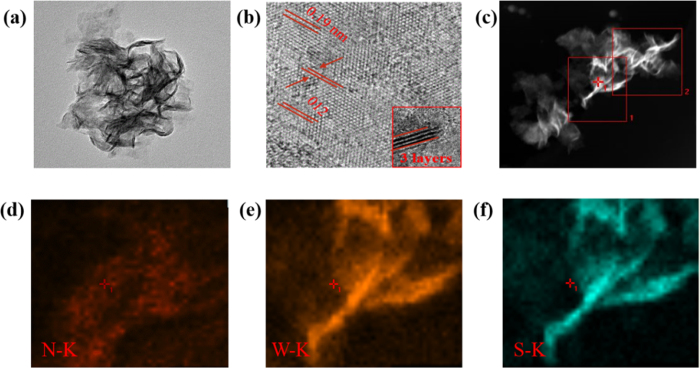
(**a**) TEM, (**b**) HRTEM, and (**c**–**f**) EDS mapping images of N-doped WS_2_ nanoflowers (S1.2).

To further verify the morphology of the as prepared sample, the TEM measurement was employed. As illustrated in Fig. 3a and c, the results also indicate our sample (S 1.2) shows the nanoflower-structure. From the high-resolution TEM (HRTEM) of N-doped WS_2_ nanoflowers (S1.2, shown in Fig. 3b), it can be intuitively seen that the sample reveals prefect lattice features, meanwhile, the interlayer spacing of ≈1.9 nm agrees well with the (012) planes of WS_2_. The inset of Fig. 3b shows the outstanding layered structure of the N-doped WS_2_ nanoflowes. Figure 3c shows the HAADF-STEM (High-angle annular dark-field scanning transmission electron microscopy) image of S1.2. In addition, N, W and S element mapping are shown in Fig. 3d to f respectively, where the result indicates N element is evenly distributed in the sample.

**Figure 4:**
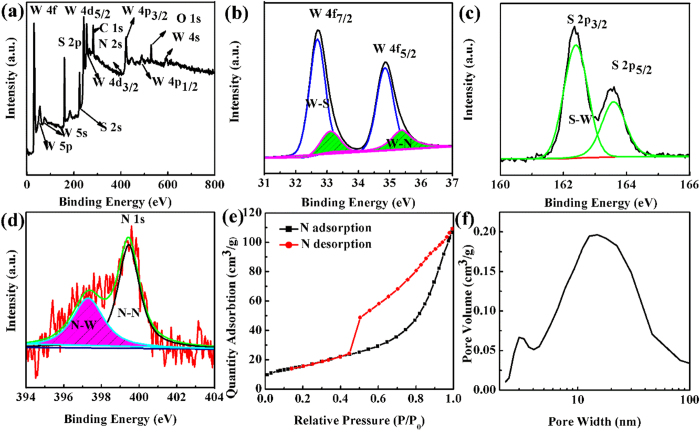
(**a**) Full range of the XPS spectrum, (**b**) XPS peaks of W 4f, (**c**) S 2p and (**d**) N 1s of N-doped WS_2_ nanoflowers. (**e**) N_2_ adsorption-desorption isotherm and (**f**) pore size distribution plot of N-doped WS_2_ nanoflowers (S1.2).

To study the composition and chemical nature of the as-prepared N-doped WS_2_ nanoflowers, XPS spectrum was exployed. As shown in Fig. 4a, it can be clearly seen that the full range XPS spectrum of the N-doped WS_2_ nanoflowers (S1.2) only contains N, S, and W elements, indicating there is no impurity elements in the sample. The high-resolution XPS spectrum of W 4f_7/2_ and W 4f_5/2_ are located at 32.7 and 34.8 eV, as shown in Fig. 4b. The XPS spectrum of W 4 f for S 1.2 can be deconvoluted into four peaks, which are attributed to the following functional groups: W-N bonds (33.2 eV and 35.3 eV) and W-S bonds (32.6 eV and 34.7 eV), indicating parts of S sites were replaced by N in WS_2_. Meanwhile, Fig. 4c shows the S 2p XPS spectrum, which can be separated into two peaks at 162.4 eV and 163.5 eV, corresponding with S-W bonds of S 2p_3/2_ and S 2p_1/2_. In order to further prove that the parts of S sites are replaced by N in WS_2_, the XPS spectrum of N 1s is fitted. As shown in Fig. 4d, two well-defined peaks can be distinguished, which indicated the N 1s binding energies were 397.4 eV and 399.5 eV, respectively. Generally, the peaks at 400 eV can be assigned to N that is surface bond with N or O, which is in agreement with other previous results^35^. Another peaks at 397.2 eV can be assigned to N-W band^36^, further indicating the parts of S sites are replaced by N on WS_2_. In addition, the nitrogen adsorption-desorption curves were performed to further study the specific surface area of the samples and the result of the representive sample S 1.2 are presented in Fig. 4e and f, revealing the sample has a larger BET area of representive sample S 1.2 (58.87 m^2^/g), which is lager than report results of Wu *et al*. (1.6 m^2^/g)^37^ and Mackie *et al*. (3.05 m^2^/g)^32^. The much enhanced surface area is beneficial for facilitating catalytic reaction in terms of the increase in the number of active sites^38^.

### Evaluation of photocatalytic Reaction

**Figure 5:**
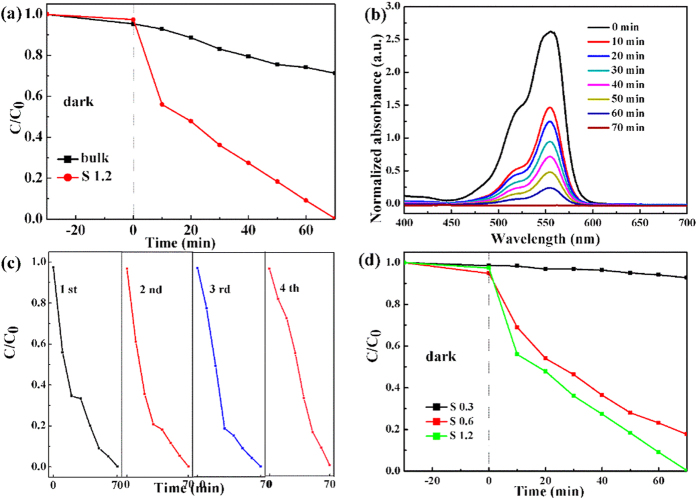
(**a**) Visible-light-promoted photocatalytic degradation of RhB carried out by S 1.2 and the bulk. (**b**) UV-vis spectra of RhB solution degraded by S 1.2 after 70 minutes (RhB solution 20 mg l^−1^). (**c**) Reusability experiment for degradation of RhB by N-doped WS_2_ nanoflowers under visible light irradiation. (**d**) Photodegradation of RhB by S 0.3, S 0.6 and S 1.2 under visible light irradiation.

The photocatalytic performances of the as-prepared samples were evaluated by degrading of RhB aqueous solution at room temperature under visible light irradiation, as shown in Fig. 5. As shown in Fig. 5a, the sample S 1.2 and its bulk were used in degrading the RhB under visible light irradiation, which can be clearly seen that the degrading rate of RhB of S 1.2 is larger than its bulk in a visible light irradiation although the absorbed rate of RhB of sample S 1.2 shows the similar value with its bulk in a dark condition (Table 1) (Supporting Information S1), which may be corresponding with its bandgap (S 1.2 1.68 eV, bulk 1.82 eV) and BET area (S 1.2 58.87 m^2^/g, bulk 24.64 m^2^/g), as shown in Figure S1 (Supporting Information). Meanwhile, plots of the absorbance versus wavelength for degradation of RhB for N-doped WS_2_ nanoflowers at various irradiation times is shown in Fig. 5b. It can be seen that the intensity of the absorption peaks continuously decreases without any changes in their position during the degradation reactions, and its intensity sharp decreases in 10 minutes and then disappear gradually in 70 minutes. For the purpose of practical use, the stability of S 1.2 was also investigated by the degradation of RhB under visible-light irradiation (Fig. 5c). It can be clearly seen that the as-prepared N-WS_2_ nanoflowers does not exhibit obvious loss in photocatalytic activity even after using for 4 cycles, revealing its excellent recycling and structural stability (previous XRD and Raman results). In addition, to study the influence of the N concentration on the photocatalytic activity of N-doped WS_2_ nanofloweres, a series of photocatalytic experiments were carried out for the N-doped WS_2_ nanoflowes with different N concentration. As can be clearly seen from Fig. 5d that the degrading rate of RhB with the photocatalysts followed the order of S 1.2 (1.2 at% N) > S 0.6 (0.6 at% N) > S 0.3 (0.3 at% N), indicating the degradation rate is gradually increases with the increasing of the N concentration.

### Mechanism of Enhanced Photocatalytic Activity and Efficiency

**Figure 6:**
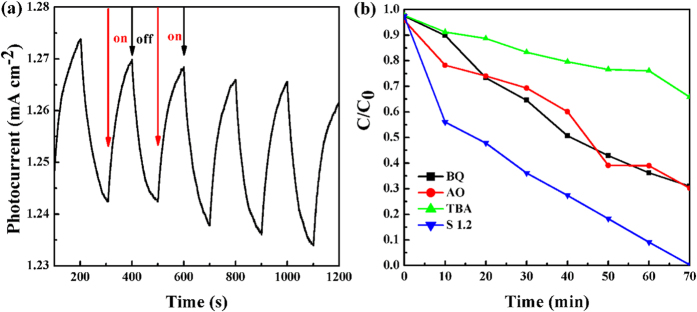
(**a**) Transient photocurrent response of S 1.2. (**b**) Photocatalytic degradation of RhB over S 1.2 in the different conditions under visible light irradiation: adding 5 ml BQ, AO, and TBA.

The separation efficiency of photogenerated electron and hole pairs plays an important role in the enhancement of photocatalytic activity, which can be confirmed by the photocurrent measurement^39^,^40^. Actually, larger magnitude of photocurrent suggests higher charge collection efficiency of the electrode surface, indicating higher separation efficiency of electron-hole pairs. Figure 6a shows the photocurrent results of sample S 1.2. Comparing with recently reported photocatalysts, such as Jia *et al*. (1.7*10^−5^ A/m^−2^)^41^, Wen *et al*. (1.75*10^−3^ A/m^−2^)^42^, Zhi *et al*. (3.2*10^−1^ A/m^−2^)^43^, Wei *et al*. (2.4*10^−5^ A/m^−2^)^44^ and Gui *et al*. (1.75*10^−1^ A/m^−2^), our sample possesses a highest photocurrent of (7.04*10^−1^ A/m^−2^), indicating the higher separation efficiency of electron-hole pairs. In order to verify which parameter of hydroxyl radical (∙OH), superoxide radical 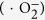, and holes (h^+^) influences the photocatalytic degradation process, the degradation of RhB over S 1.2 with various scavengers were explored. As shown in Fig. 6b, for our N-doped WS_2_ system, the photocatalytic performance decreased greatly by addition of TBA or *t*-BuOH (Supporting Information S4), but changed very slightly by addition of others scavengers, suggesting that the hydroxyl radical is the domination oxidative species of N-doped WS_2_ and others only play an assistant roles.

**Figure 7:**
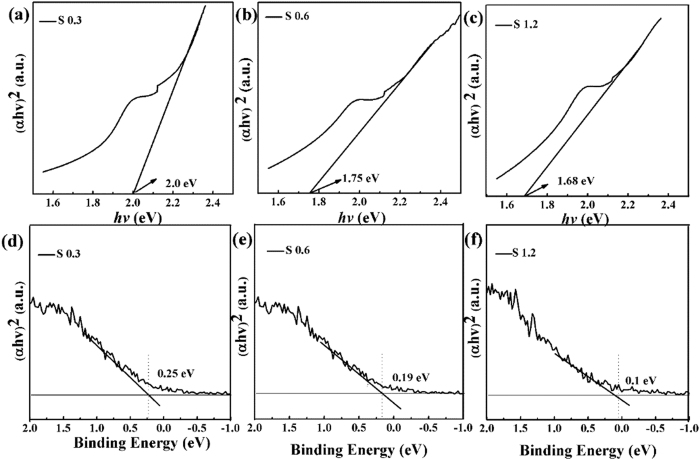
(**a–c**) UV-DRS spectrum of S 0.3, S 0.6 and S 1.2. (**d,e**) Valence-band XPS spectra of S 0.3, S 0.6 and S 1.2.

The band-gap energy of all the samples are estimated from the plot of (*ahv*)^n^ versus *hv* by extrapolating the straight line to the *X* axis intercept, as shown in Fig. 7a–c. The band-gap energies of S 0.3, S 0.6 and S 1.2 are found to be 2.0, 1.75 and 1.68 eV, respectively. Results indicate the band-gap energy is gradually decreased with the increasing of the N concentration^45^. In addition, to further study the influence of the N concentration on the band gap, the density of states (DOS) of the valence band of N-doped WS_2_ photocatalysts were measured by the valance band XPS. As shown in Fig. 7d–f, it can be clearly seen that the edge of the valance band energy with the photocatalysts followed the order of S 0.3 > S 0.6 > S 1.2, indicating the valance band maximum rise with low density of states^46^. The band gap shift is attributed to lattice defects such as those arising from interstitial nitrogen^47^.

**Figure 8:**
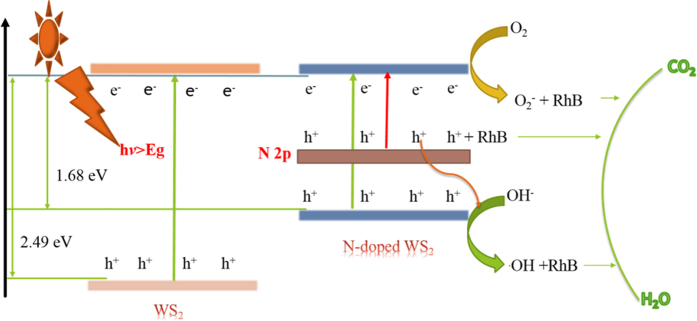
The mechanism of charge separation and photoactivity of WS_2_ and N-doped WS_2_ nanoflowers under visible light irradiation.

Based on the above results, a schematic diagram for the density of states of pure WS_2_ and N-doped WS_2_ nanoflowers has been proposed shown in Fig. 8 to give the mechanism of enhanced Photocaptalytic activity and efficiency in N doped WS_2_ nanoflowers. The forbidden gap of pure WS_2_ (2.49 eV) was reported by Hong *et al*.^48^, which can only absorb light wavelength less than 498 nm. In recent reports, numerous investigations have been enhanced photocatalysis efficiency by introduced surface oxygen vacancies in several semiconductors, such as, BiPO_4_^49^, CeO_2_^50^ and Bi-component Cu_2_O-CuCl^51^, which could be demonstrated to be conductive to band gap narrowing and photoactivity. Compared to the surface oxygen vacancies, the introduction of surface sulfur vacancies by doping N in our research narrows band gap and many shallow surface sulfur vacancies appear at the valance band (VB), as well as, N doping could introduce an impurity band. Furthermore, the introduction of surface sulfur vacancies can expand the VB width, which contributes to increasing the separation efficiency of photoinduced electron-hole pairs, leading to enhancement of photocatalytic activity. Moreover, N doping can extend the visible light absorption edge and the electrons are excited from the N impurity level to the conduction band, guaranteeing higher activity in degrading RhB^52^. Therefore, our sample possesses a high photocatalytic efficiency.

**Figure 9:**
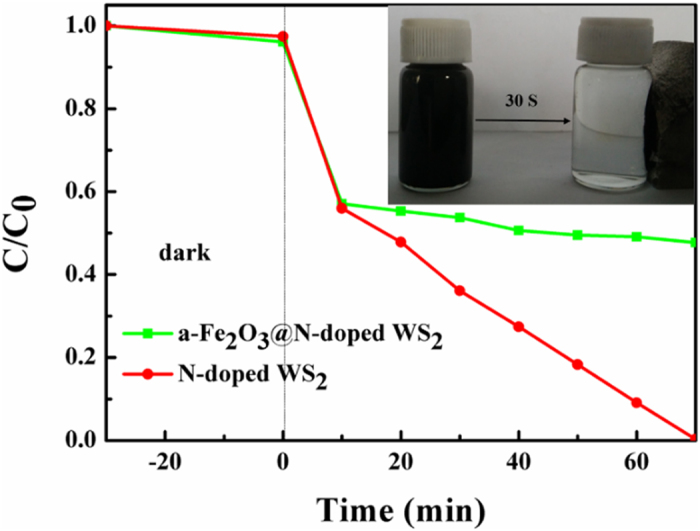
Photocatalytic degradation of RhB by N-doped WS_2_ nanoflowers (S1.2) and a-Fe_2_O_3_ heterostructure under visible light irradiation. Inset shows the magnetic separation.

In addition, although as-prepared N-doped WS_2_ nanoflowers show obvious photocatalysis, it is not so easy to recycle. Catalysts with magnetic properties, namely magnetic catalysts could overcome this problem^53^. Therefore, it is gratifying to find a strategy for fabricating magnetic photocatalysts. Recently, magnetically separable semiconductor materials have attracted increasing attention because of their efficient recycle in water treatment, such as, Ni-Au-Zn^54^, NiO nanosheets^55^, Ag@AgCl^56^, r-Fe_2_O_3_@TiO_2_^57^and etc. Here, α-Fe_2_O_3_@N-doped WS_2_ heterostructure with strong magnetic property was prepared and employed to magnetically separate our catalysts from the solution of RhB. The SEM and TEM results of α-Fe_2_O_3_@N-doped WS_2_ heterostructure is shown in Figure S3 (Supporting Information). As shown in Fig. 9, the degradation rate of RhB is almost 50% in 70 minutes, and it can be magnetically separation in 30s (shown in the upper right of Fig. 9). These results indicate that α-Fe_2_O_3_@N-doped WS_2_ heterostructure can not only serve as efficient photocatalysts but also easy separate from organic pollutants.

## Conclusions

In summary, we fabricated a series of W(N_x_S_1−x_)_2_ nanoflowers via regulation of the mass ratio between tungsten pentachloride and thiourea in a mixed solvent system, as well as, fabricated the α-Fe_2_O_3_@N-doped WS_2_ heterostructure. Under visible light irradiation, N doping can significantly increase the photocatalytic performance of WS_2_ with the best efficiency obtained for 1.2 at% nitrogen doping. The expanded the utilization of visible light and the enhanced photoccatalytic activity both are resulted from the production of the surface sulfur vacancies by N doping. Meanwhile, we also demonstrated that the α-Fe_2_O_3_@N-doped WS_2_ heterostructure can be easily separated from the organic pollutants, which improves the actual utilization rate of our sample.

## Additional Information

**How to cite this article**: Liu, P. *et al*. Efficient visible light-induced degradation of rhodamine B by W(N_x_S_1−x_)_2_ nanoflowers. *Sci. Rep.* **7**, 40784; doi: 10.1038/srep40784 (2017).

**Publisher's note:** Springer Nature remains neutral with regard to jurisdictional claims in published maps and institutional affiliations.

## Supplementary Material

Supplementary Information
