# Supplementary material for: Efficient visible light-induced degradation of rhodamine B by W(NxS1−x)2 nanoflowers
Source: Sci Rep. 2017 Jan 20;7:40784. doi: 10.1038/srep40784 (PMC5247754; doi:10.1038/srep40784)
Supplement: Supplementary Information [file srep40784-s1.docx]

**Efficient visible light-induced degradation of rhodamine B by W(N_x_S_1-x_)_2_ nanoflowers**

Peitao Liu^a^, Jingyan Zhang^a^, Daqiang Gao^a*^ and Weichun Ye^b*^

**^a^** *Key laboratory for magnetism and Magnetic Materials of MOE, Lanzhou University, Lanzhou 730000, P. R. China.*

**^b^** *Department of Chemistry, Lanzhou University, Lanzhou 730000, P. R. China.*

*Corresponding email:* [*gaodq@lzu.edu.cn*](mailto:gaodq@lzu.edu.cn)*,* [*yewch@lzu.edu.cn*](mailto:yewch@lzu.edu.cn)*.*

*
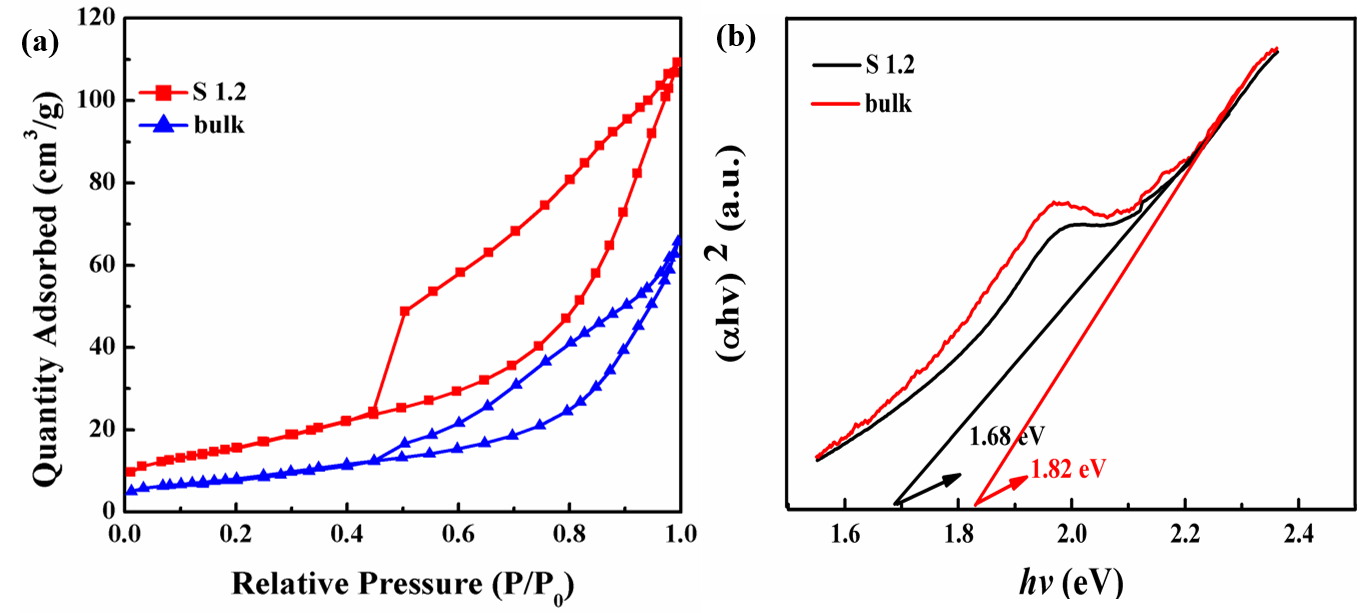
*

*

*S 1. (a)-(b) The measured BET area and estimated the bandgap for sample S 1.2 and its bulk form.

S 2. XRD pattern of 3 g CH_4_N_2_S and 0.5 g WCl_6_.


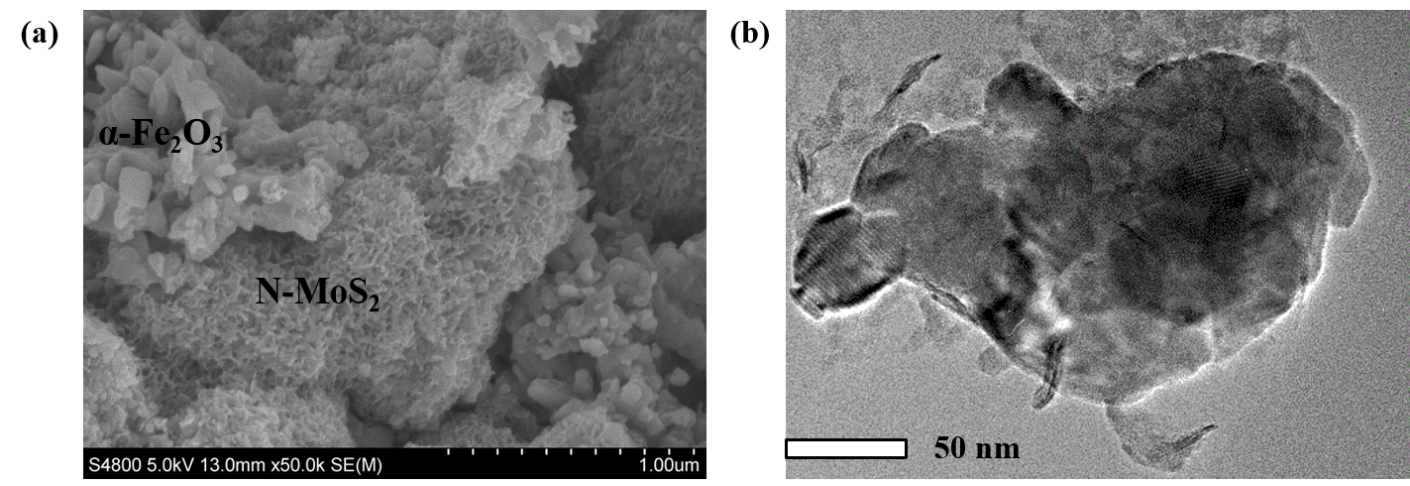


S 3. SEM and TEM image of α-Fe_2_O_3_@N-doped WS_2_.

*
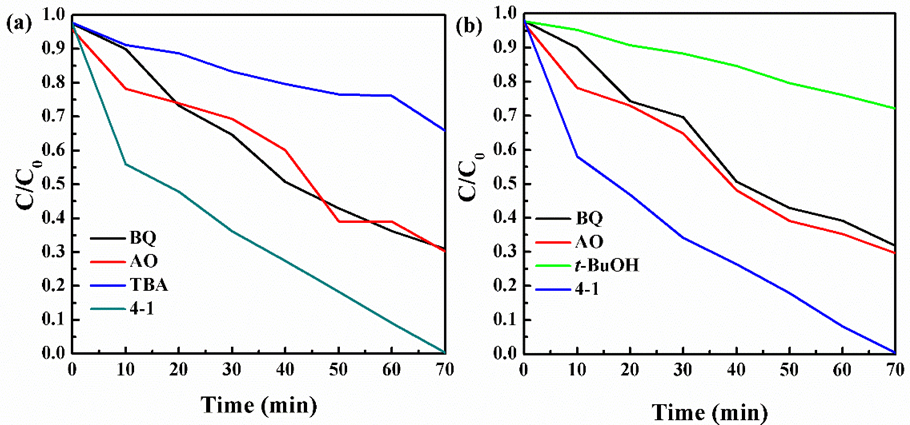
*

S 4. (a)-(b) Photocatalytic degradation of RhB over S 1.2 in the different conditions under visible light irradiation: adding BQ, AO, TBA and *t*-BuOH (0.1 g/L).

| samples | C/C_0_ |
| --- | --- |
| bulk | 0.952±0.035 |
| S 1.2 | 0.986±0.015 |

Table 1. The C/C_0_ (the initial concentration *C_0_*. *C* the actual concentration of RhB at reaction time) of sample S 1.2 and its bulk form after 30 minutes in a dark condition.

**Acknowledgment**

This work is supported by National Basic Research Program of China (Grant No. 2012CB933101), the National Natural Science Fundation of China (Grant No. 11474137, 51301081 and 11274146), and the Fundamental Research Funds for the Central Universities (Grant No.lzujbky-2014-27 and No.lzujbky-2016-130).

**Author Contribution statement**

Peitao Liu and Daqiang gao wrote the main manuscript text and prepared figures 1.2.3.4.5.6.7.8.and 9. Jingyan Zhang and Weichun Ye reviewed the manuscript.

**Addition Information**

**Supplementary information** accompanies this paper at <http://www.nature.com/srep>

**Competing financial interests:** The authors declare no competing financial interests.
